# Recalibration of mapping quality scores in Illumina short-read alignments improves SNP detection results in low-coverage sequencing data

**DOI:** 10.7717/peerj.10501

**Published:** 2020-12-07

**Authors:** Eliot Cline, Nuttachat Wisittipanit, Tossapon Boongoen, Ekachai Chukeatirote, Darush Struss, Anant Eungwanichayapant

**Affiliations:** 1 School of Science, Mae Fah Luang University, Amphur Muang, Chiang Rai, Thailand; 2 Department of Biotechnology, East West Seed Company, San Sai, Chiang Mai, Thailand; 3 Center of Excellence in AI and Emerging Technologies, School of Information Technology, Mae Fah Luang University, Amphur Muang, Chiang Rai, Thailand

**Keywords:** Machine learning, Low coverage sequencing, Variant calling, Probability calibration, BAM, Genome simulation

## Abstract

**Background:**

Low-coverage sequencing is a cost-effective way to obtain reads spanning an entire genome. However, read depth at each locus is low, making sequencing error difficult to separate from actual variation. Prior to variant calling, sequencer reads are aligned to a reference genome, with alignments stored in Sequence Alignment/Map (SAM) files. Each alignment has a mapping quality (MAPQ) score indicating the probability a read is incorrectly aligned. This study investigated the recalibration of probability estimates used to compute MAPQ scores for improving variant calling performance in single-sample, low-coverage settings.

**Materials and Methods:**

Simulated tomato, hot pepper and rice genomes were implanted with known variants. From these, simulated paired-end reads were generated at low coverage and aligned to the original reference genomes. Features extracted from the SAM formatted alignment files for tomato were used to train machine learning models to detect incorrectly aligned reads and output estimates of the probability of misalignment for each read in all three data sets. MAPQ scores were then re-computed from these estimates. Next, the SAM files were updated with new MAPQ scores. Finally, Variant calling was performed on the original and recalibrated alignments and the results compared.

**Results:**

Incorrectly aligned reads comprised only 0.16% of the reads in the training set. This severe class imbalance required special consideration for model training. The F1 score for detecting misaligned reads ranged from 0.76 to 0.82. The best performing model was used to compute new MAPQ scores. Single Nucleotide Polymorphism (SNP) detection was improved after mapping score recalibration. In rice, recall for called SNPs increased by 5.2%, while for tomato and pepper it increased by 3.1% and 1.5%, respectively. For all three data sets the precision of SNP calls ranged from 0.91 to 0.95, and was largely unchanged both before and after mapping score recalibration.

**Conclusion:**

Recalibrating MAPQ scores delivers modest improvements in single-sample variant calling results. Some variant callers operate on multiple samples simultaneously. They exploit every sample’s reads to compensate for the low read-depth of individual samples. This improves polymorphism detection and genotype inference. It may be that small improvements in single-sample settings translate to larger gains in a multi-sample experiment. A study to investigate this is ongoing.

## Introduction

High-throughput DNA sequencing data is popular due to its low-cost and the availability of tools for processing and analysis. Sequencers produce short “reads” between hundreds to thousands of base pairs (bp) long. Reads aligned to a reference genome assembly are used to call variants, including single nucleotide polymorphisms (SNP), small insertions and deletions (INDEL) and larger, structural variants (SV). Software exists that accurately aligns reads to a reference genome. However, sequencing errors, repetitive regions, inserts and deletions can cause aligners to place reads incorrectly, or not at all.

Alignment software assigns reads a Mapping Quality (MAPQ) score representing the negative, log-scaled probability that a read is misaligned. High scores indicate high confidence that a read is aligned correctly, while low scores indicate the opposite. MAPQ scores are often presented as *Q_m_* = −10*log*_10_(*p*), where *p* is the misalignment probability. Methods of estimating *p* vary among alignment programs and may account for the number of mismatches and INDELs in a read or the number of times a read aligns to its reference. MAPQ scores are influential on the outcome of variant calling as higher quality alignments generally lead to higher confidence calls. Variant calling is also influenced by depth of coverage. Generally, coverage in the range of 20× to 30× is necessary to capture 90% of all SNPs between two samples of organisms with human-sized genomes (Sims et al., 2014).

Most variant calling programs operate by examining aligned reads and comparing base calls at each position with the corresponding reference base. If a read’s base differs from the reference, the software calculates the probability that this difference does not represent a true variant. The calculation considers parameters such as coverage, base call quality and proximity to INDELs. This probability is used to assign a quality score to each variant that is the log-scaled probability that the call is wrong.

While sequencing costs have dropped recently, sequencing the genomes of entire populations at high coverage can still be prohibitively expensive. Genotyping by Sequencing (GBS) is used to sample an organism’s genome. This “complexity reduction” approach is popular as it is inexpensive and delivers many thousands of SNPs genome wide. However, GBS does not deliver consistent results from sample to sample and is prone to large amounts of missing data, the under-calling of heterozygotes and low SNP density (Negro et al., 2019).

Variant calling with Whole Genome Shotgun (WGS) sequencing at low-coverage (<5×) yields high SNP density, but suffers from high false positive rates and heterozygote under-calling (Yu & Sun, 2013). Variant calling packages such as FreeBayes (Garrison & Marth, 2012), Bcftools (Li, 2011) and GATK (DePristo et al., 2011) can operate on multiple samples simultaneously. They exploit every sample’s reads to compensate for the low coverage of individual samples, improving polymorphism detection and genotype inference.

A previous study (Ruffalo et al., 2012) claimed that MAPQ scores reported by read aligners do not correlate well with accuracy. They applied a logistic regression model to re-calibrate the probability estimates used to compute MAPQ scores and claimed that this improved SNP calling results. Another study (Yang et al., 2015) used Support Vector Machines (SVM) to identify misaligned reads and filter them out of the data set. They also claimed improved SNP calling results.

Both studies relied on simulated data to evaluate the effectiveness of their respective approaches. Varying numbers of artificial SNPs were implanted into human genome sequences from which simulated reads were generated. The reads were then aligned to the original reference genome and SNPs were called. With this approach we can know the correct locations (with respect to the reference genome) of each SNP and simulated read. Accurate comparisons of SNP calling and read mapping results can be made before and after the application of probability re-calibration or read filtering.

In our study we simulated genome assemblies for tomato, pepper and rice by implanting known variants into their respective published, reference assemblies. Simulated reads generated from these assemblies were aligned to the original genomes. We investigated how accurately machine learning models could identify misaligned reads. The models output probability estimates that each read was misaligned. These were used to re-calibrate MAPQ scores and update the original alignment files. Finally, SNPs were called before and after MAPQ score re-calibration to determine if results could be improved in a single sample, low-coverage setting.

This study differs from previous studies in several ways. Previous studies implanted only SNPs into the assemblies used as a base for simulating reads. In real, biological samples we find large numbers of SNPs, INDELs and SVs, even between closely related samples. The first goal of this study was to create more realistic simulated genomes by implanting known variants of multiple types. Previous studies simulated portions of the human genome, whereas we simulate the complete genomes of three plant species. Other studies tested only a single machine learning model trained on high-coverage data. Here, the effects of multiple models trained on low-coverage data, and multiple data sampling approaches were examined. We evaluated the effects of recalibrating MAPQ scores on SNP discovery using two variant calling packages. The procedure was also tested on real cucumber sequencing data. Program source code, links to data sources and usage instructions are available at https://github.com/bioinfo2019/qual-recal.

## Materials and Methods

The starting point for this study was the simulation of diploid, eukaryotic genomes containing known variants. Simulated reads were generated from these simulated genomes and used in subsequent steps in this analysis.

### Reference genomes and variants

Simulation starts with implanting a set of variants into a reference genome. While we cannot expect to perfectly capture an organism’s genome in a simulation, we can take steps to ensure that our simulation mimics reality as closely as possible. The organisms selected for simulation in this study were tomato, pepper and rice. Genome assemblies, and sets of SNPs and INDELs, are available for all three.

#### Tomato

The reference genome used was the version SL2.50 assembly of the *Solanum lycopersicum*, Heinz 1706 inbred cultivar (The Tomato Genome Consortium, 2012). It is approximately 900 Mb in size and has 12 chromosomes containing roughly 35,000 genes.

Sixty recombinant inbred lines (RIL) of *S. lycopersicum* were sequenced at 12× coverage as part of the 150 Tomato Genomes project (Aflitos et al., 2014). Reads from 14 of the RILs were used to call SVs with the SVDETECT software (Zeitouni et al., 2010). Variants shared by all 14 accessions were retained, totaling over 10,000 large (>1 kbp) deletions, duplications, inversions and translocations. The project includes variant calls for the RILs. One set of approximately 270,000 SNPs and INDELs called between the *Solanum lycopersicum* cv. Ailsa Craig and the Heinz 1706 reference was used to create the simulated tomato genome.

#### Chili pepper

The pepper genome assembly was derived from sequencing the Mexican landrace of *Capsicum annuum* cv. CM334 (Kim et al., 2014), version 1.55. It is also a member of the Solanaceae family and has 12 chromosomes containing a similar number of genes. Genome size is around 3.48 Gb with 3.06 Gb contained in the assembly.

SNPs and INDELS were called between the reference genome and the Korean, inbred line *Capsicum annuum* cv. Taean (Kang et al., 2016). Of a total of over six million variants, a random selection of approximately 288,000 were used in the simulation.

#### Rice

The rice assembly is the Chinese subspecies, *Oryza sativa* L. ssp. *indica* (Yu et al., 2002), version ASM465v1. It is 466 Mb long, has 12 chromosomes and 10,454 unanchored contigs. Only the 12 chromosomes were used for the simulation.

A set of approximately 35,000 SNPs and INDELs was implanted into the simulated rice genome. The SNPs and INDELs were called between the reference genome and the *Oryza sativa* L. *Japonica* cv. Nipponbare (Zhao et al., 2004).

#### Cucumber

The cucumber assembly was created from sequencing the “Chinese Long” variety, line 9930, *Cucumis sativus* L. var. *sativus* cv. 9930 (Li et al., 2019). The version 3.0 genome sequence is 260 Mb in length, has seven chromosomes and 78 unanchored contigs.

Reads for the Chinese Long variety were obtained from the NCBI Sequence Read Archive, accession number PRJNA339498. The reads are 101 bp in length and were generated by the Illumina HS2000 sequencer, the same sequencer simulated in this study. These reads were used to test the models developed with simulated data.

### Diploid genome simulation

Variants were implanted into the three reference genomes using the Varsim package (Mu et al., 2015). Varsim simulates diploid genomes and Illumina sequencing reads of varying lengths. It takes a reference genome and lists of variants as inputs and outputs a diploid genome with the variants implanted in it. VarSim employs the ART (Huang et al., 2012) Illumina read simulator to generate reads at a specified coverage. These reads contain base call errors and very small INDELs. The number and distribution of the errors are modeled on error profiles from actual sequencing instruments. Varsim modifies the resulting read names to include the reference genome coordinates of a read’s origin. Therefore, we can know exactly where in the reference genome a read originated and whether it aligned to its correct location. Figure S1 shows the simulation process using VarSim.

Paired-end reads at 100 base pairs in length were generated from the simulated tomato genome and aligned to the original reference assembly. Sequencing depth was set to 3× and mean fragment size was set to 240 bp with a standard deviation of 80 bp. These settings closely mirror the fragment length mean and standard deviations in the 150 Tomato Genomes RIL data. Features for training machine learning models were extracted from the alignments of the simulated tomato reads. The rice and pepper genomes were used to generate test data to validate the models.

### Read mapping

Read alignment was performed with the Bowtie2 software using default parameters (Langmead & Salzberg, 2012). Bowtie2 is a fast, accurate aligner capable of mapping millions of reads to large reference genomes in a short amount of time. Bowtie2 aligned just over 99% of the simulated reads to the reference genomes.

### Feature engineering

Some of the features in our study were used in the cited studies. Others were calculated from properties of reads or reference sequences, while others were specific to the Bowtie2 aligner (Table S1).

Several of them originate from the Sequence Alignment/Map (SAM) files produced by Bowtie2 (for more information, refer to the SAM format specification (Li et al., 2009)). The features—SLOPE, INTERCEPT and R_VALUE—were calculated by computing the least-squares line of a read’s base quality scores against their positions in the read. R_VALUE is the Pearson correlation between base quality and position in the read sequence. These features were used in the study by Ruffalo et al. (2012).

**Figure 1:**
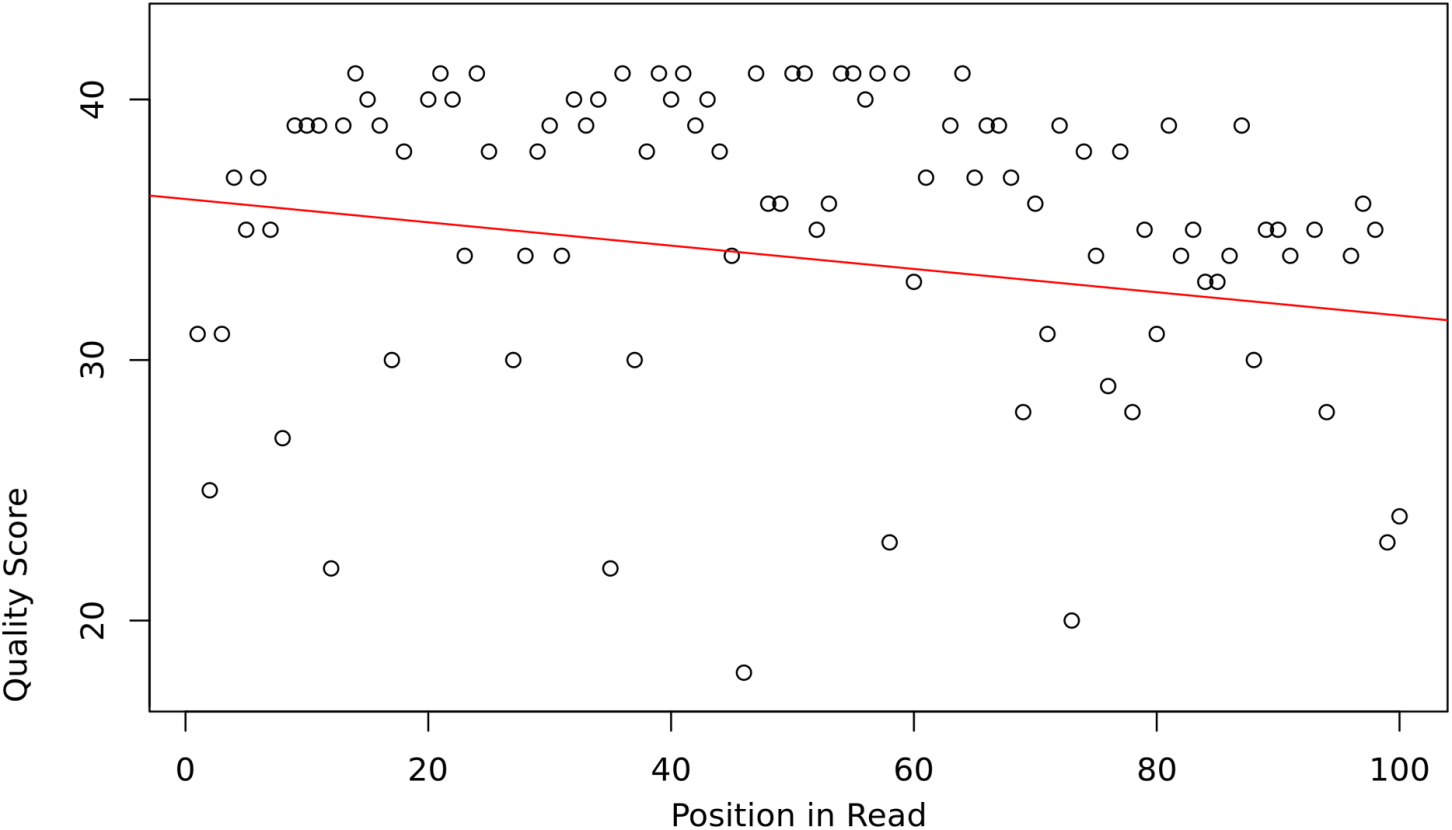
Base quality vs position in a read.

The assumption is that the slope and intercept serve as a proxy for overall read sequence quality, and that these measures can capture the position-dependent nature of base call qualities in Illumina sequencing reads, as they tend to contain more low-quality bases at the beginning and end of reads (Fig. 1).

Plant genomes contain large amounts of repetitive sequence. In repetitive regions, a read may map to multiple locations equally well. Bowtie2 reports a score for each alignment, and a score for the best secondary alignment. Thus, if ALIGN_SCORE is equal to SECONDARY_ALIGN_SCORE, the aligner placed the read equally well in at least two locations. Bowtie2 assigns these reads a MAPQ score of 1. These “multi-mapped” reads were excluded from the training and testing data.

Some features are intrinsic to the read or reference sequence, such as GC content. READ_GC_CONT is the GC content of a read and REF_GC_CONT is the GC content of the region of the genome the read maps to. The feature READ_COMPLEXITY_SCORE is the size of the read sequence after compressing with Unix gzip. Similarly, REF_COMPLEXITY_SCORE is the compressed size of the region in the genome to which the read maps. Generally, text with many repeated words and phrases will have a smaller compressed size than an equivalent number of random letters. These features are meant to characterize the amount of repetitive content in the sequences.

### Feature selection

We used two feature selection methods, Information Gain and ReliefF. Their implementations are found in the machine learning platform Orange3 (Demšar et al., 2013). Figure S2 details the feature selection process. Starting with the aligned reads in binary SAM (BAM) format, features were extracted and each vector was assigned to Class 0 (correctly aligned) or Class 1 (incorrectly aligned). To create the feature selection data set, all Class 1 reads were combined with an equal quantity of randomly selected Class 0 reads. This subset was used with Information Gain and RefliefF to select the final feature set.

Both Information Gain and ReliefF are algorithms that try to rank features by their contribution to class separability. Information Gain is a measure of the decrease in information entropy when a particular feature is used to classify a data set. ReliefF uses a nearest neighbor classifier to compute a score for each feature. The higher the score, the more informative the feature.

A total of 22 features were extracted or computed from the BAM files after read mapping. Seven of these features were found to be informative. Table 1 shows all 22 features’ scores for both Information Gain and ReliefF. A thin black line separates the seven top-ranked features. Note the sharp decrease in values of both Information Gain and ReliefF from the seventh to the eighth feature. This was chosen as the cutoff for the final selection.

**Table 1:** Feature importance rankings sorted by Info Gain.

Feature	Info Gain	ReliefF
MAPPING_QUALITY	0.588	0.098
ALIGN_SCORES_DIFF	0.460	0.181
EDIT_DISTANCE	0.418	0.054
ALIGNMENT_SCORE	0.417	0.114
MISMATCHES	0.407	0.0470
SECONDARY_ALIGN_SCORE	0.379	0.248
MATE_ALIGN_SCORE	0.206	0.091
GAP_OPENS	0.072	0.013
GAP_EXT	0.071	0.008
PAIR_ALIGN_TYPE	0.052	0.01
INSERT_SIZE	0.051	0.001
REF_GC_CONT	0.042	0.022
READ_GC_CONT	0.026	0.025
PAIR_ORIENTATION	0.014	0.0004
N_LOWQ_BASES	0.001	0.03
REF_COMPR_SIZE	0.001	−0.001
READ_COMPR_SIZE	0.001	0.005
SLOPE	0.001	0.01
AVG_LOWQ_SCORE	0.0004	0.007
INTERCEPT	0.0004	0.012
R_VALUE	0.00038	0.014
DEPTH	0.00033	0.027

### Training and classification

A C++ program was written to compute and extract features from the Bowtie2 alignment files. The feature vector values were scaled from 0.0 to 1.0 using the minmaxscaler in the *sklearn* Python package (Pedregosa et al., 2011). This transformation is necessary as many of the features are of very different numerical scales. For the tomato data set, in an alignment of just over 22 million reads, approximately 36,000 were found to be misaligned. The proportion of misplaced reads ranged from 0.14% to 0.19% of the total reads for all three organisms, resulting in an extreme imbalance between classes. Standard machine learning algorithms tend to perform badly with severely imbalanced class distributions. In this situation the simple notion of classification accuracy is not an effective measure of classifier performance. For instance, if we were to simply classify all reads as belonging to Class 0, overall accuracy would still be better than 99%.

Many algorithms have been developed to deal with imbalanced class distributions. Re-sampling the training data is a popular approach. Under-sampling removes samples from the majority class. In its simplest form, majority-class samples are randomly removed until the number of samples remaining is roughly equal to the number of minority-class samples. A classifier is then trained on the balanced sample. The major problem with this approach is that important samples in the majority class may be discarded. Over-sampling generates random copies of the minority-class samples, or generates synthetic, minority-class examples in sufficient quantities to balance the training set.

A classifier can be trained on a single random sample, but the resulting model may not generalize well to unseen samples. Another approach would be to train a classifier on all possible balanced subsets of the data and select the best performing model. Two popular approaches are bagging and boosting. Bagging is short for bootstrap aggregation. The idea is to train a classifier on all, or many, random subsets of the training data and aggregate the results into a final predictive model. Boosting is similar except that models are trained sequentially. In each iteration misclassified samples are given higher weights than correctly classified samples so that at the next iteration they may be classified correctly. Both boosting and bagging methods are called ensemble methods as they combine multiple models to come up with a final model.

In this article several models and sampling schemes were tested. Results of each were compared using performance metrics appropriate for the severe class imbalance present in the data. The metrics used were F1 score, average precision score and the Brier score.

(1) $${\rm Precision} = {\rm TP}/({\rm TP} + {\rm FP})$$

(2) $${\rm Recall} = {\rm TP}/({\rm TP} + {\rm FN})$$

(3) $${\rm F1} = 2*({\rm Precision}*{\rm Recall}/({\rm Precision} + {\rm Recall}))$$

(4) $${\rm BS} = \displaystyle{1 \over N}\sum\limits_{t = 1}^N {({f_t} - {o_t})^2}{\kern 1pt}$$

(5) $${\rm AP} = \sum\limits_n ({R_n} - {R_{n - 1}}){P_n}$$

The F1 score, in Eq. (3), is the harmonic mean of Precision and Recall. Eqs. (1) and (2) show how Precision and Recall are calculated in terms of true positive (TP), false positive (FP) and true negative (TN) predictions. A perfect score of 1.0 indicates zero classification error while a score of 0.0 indicates no correct classification of the target class.

The Brier score (BS) in Eq. (4) computes the mean squared error between predicted outcomes and their probability of occurrence. The Brier score is a measure of how well calibrated a set of probabilistic predictions is. For this study, accurate probability estimates were needed to re-estimate MAPQ scores. In Eq. (4), *N* is the number of predictions, *f*_*t*_ is the probability of the target class membership for the *t*_*th*_ prediction and *o*_*t*_ is the actual outcome for the *t*_*th*_ prediction. In the binary case *o*_*t*_ takes values of either 0 or 1. The Brier score attains a maximum value of 1.0 and minimum of 0.0, which indicates no classification error and theoretically perfectly calibrated probability estimates.

The Average Precision (AP) score is shown in Eq. (5). The AP score provides a single-number summary of the precision-recall curve. The formulation presented is found in the *sklearn* machine learning Python package. It computes the weighted mean of precisions achieved at each probability (or other score) threshold. For instance, when using logistic regression as a classifier a probability of greater than 50% is often used to assign a sample to the positive class. Other thresholds can be used to attain a different balance between precision and recall. In Eq. (5), *P*_*n*_ and *R*_*n*_ are the precision and recall attained at the *n*_*th*_ threshold.

Models tested include the SVM using the Radial Basis Function (RBF) kernel, Logistic Regression (LR), Decision Tree (DT) and AdaBoost. Table 2 summarizes how each model was paired with Random Under Sampling (RUS), bagging or boosting.

**Table 2:** Algorithms used in the study.

ID	Algorithm	Description
Random Under Sampling Methods		
RUSVM	SVM	SVM with RBF kernel trained on all balanced
		subsets of training data. Retain best performing model
RULR	LR	LR trained on all balanced subsets of training data
		Retain best performing model
RUSVMS	SVM	SVM with RBF kernel trained on all balanced
		subsets of training data. TP/FP rates calculated at each
		iteration and model re-trained on only TP/FP samples
RULRS	LR	Logistic regression model trained on all balanced
		subsets of training data. TP/FP rates calculated at each
		iteration and model re-trained on only TP/FP samples
Ensemble Methods		
DTBAG	DT	Balanced subset bagging using decision
		tree as the base estimator
ADBST	AdaBoost	Boosting using the classic AdaBoost algorithm
		with a decision stump as the base estimator
Hybrid Methods		
RUBST	RUSBoost	Random under sampling combined with AdaBoost

All models were trained on 20% of the 22 million read tomato data set and tested on the remaining 80%. Models were trained only on the simulated tomato data. The rice and pepper data were held out as additional test sets. Grid search with five-fold cross-validation was used to identify optimal model parameters, with F1 score used as the performance metric inside the cross-validation loop. Table S2 shows the parameters used for each model. Training example totals for both classes for all models is shown in Table S3.

Figure S3 illustrates the random under-sampling procedure. Random subsets of the training data were drawn until the number of subsets was roughly equal to the number of training examples divided by the number of minority class samples. For the tomato training data, this totaled just over 300 subsets. Each subset comprised all misaligned reads and an equal number of randomly-selected correct alignments. At each iteration, a model was trained and saved to disk. Afterwards the models were evaluated and the best one was retained for future use.

For RUSVM and RULR, models were trained on each subset and the best performer selected for prediction on the 80% test data. The two methods called RULRS and RUSVMS are similar, except that after the models were trained on a subset, predictions were made on the 20% training data and true and false positives identified. A new training set was constructed containing all false positive samples and an equal number of true positives. Training was carried out again on this new set using grid search with five-fold cross-validation. After training, prediction was done on the 80% test data. After all subsets were exhausted the best performing model was retained for later use (Fig. S4).

### Mapping quality recalibration and variant calling

After training, all selected models were used to predict misplaced reads in the 80% test data and to compute estimates of each read’s probability of misalignment. Isotonic regression was then applied to these estimates. Isotonic regression can improve the calibration of a set of probability estimates. The isotonic regression model was itself trained using probability estimates calculated from each model’s predictions on the 20% training data.

Isotonic regression fits a non-decreasing function to data. It minimizes

(6) $$\sum\limits_i {w_i}{({y_i} - {\hat y_i})^2}$$

subject to

(7) $${\hat y_{min}} = {\hat y_1} \le {\hat y_2} \ldots \le {\hat y_n} = {\hat y_{max}}$$

where *y*_*i*_ is an arbitrary real number and *w*_*i*_ is a strictly positive number. The output is a vector of numbers adjusted such that they are strictly increasing from *y*_*min*_ to *y*_*max*_. In the case of generating probability estimates *y*_*min*_ = 0 and *y*_*max*_ = 1. We used the implementation in the *sklearn* Python package.

**Figure 2:**
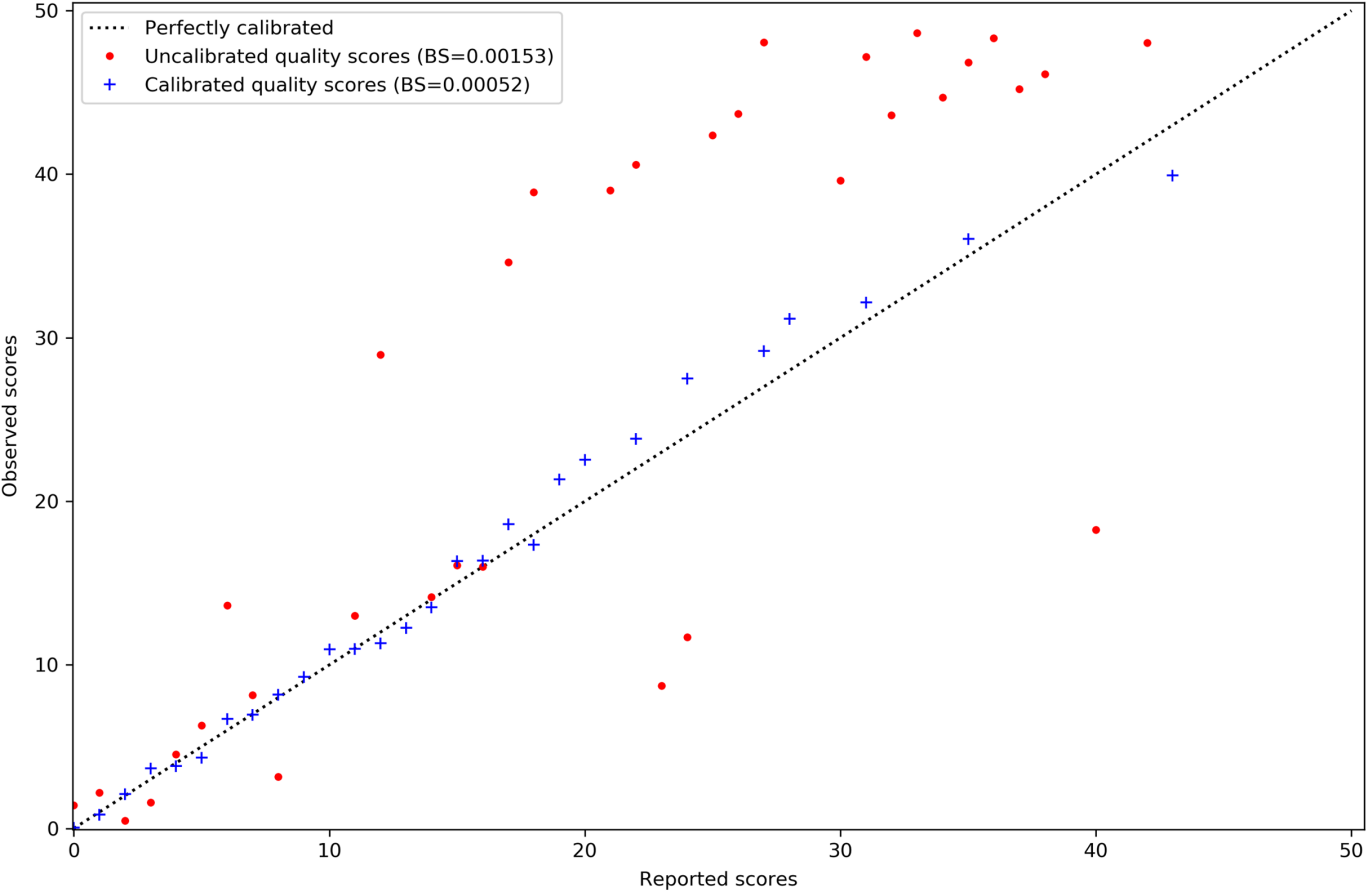
MAPQ scores for the simulated tomato data set before and after calibration. Red points are the original Bowtie2 MAPQ scores. Blue points are the scores after re-calibration. Their Brier scores are shown in the legend. Perfectly calibrated scores will fall on the diagonal line. The *x*-axis shows the MAPQ scores assigned to reads in the SAM file. The *y*-axis shows the actual, observed score. The observed score is calculated by taking the proportion of reads assigned to the positive class for a given score to the total number of reads for that score and using the proportion as *p* in *Q_m_* = −10log10(*p*).

Calibration of MAPQ scores is improved after predicting misalignment probability with AdaBoost and applying isotonic regression. The reported scores on the *x*-axis of Fig. 2 are those assigned to the misaligned reads in the simulated tomato data. Their distribution has also been shifted to the lower end of the scale. This is sensible as we would expect incorrect mappings to have lower MAPQ scores.

Performance metrics were calculated for each model before and after probability re-calibration. The top two performing models were used to predict misaligned reads on the 80% tomato data withheld as the test set. The best performing model of the top two was chosen to re-calibrate the MAPQ scores in the alignments of tomato, rice and pepper reads. FreeBayes and Bcftools were used to call variants for all three data sets using both the original and re-calibrated alignment files. A utility program included with VarSim identified the true and false positive SNP calls for all three data sets.

## Results

The percentage of misaligned reads for each organism shows class imbalance is consistent for all three (Table 3).

**Table 3:** Correctly aligned and incorrectly aligned reads per organism.

Organism	Correct	Incorrect	Pct Incorrect (%)
Tomato	21,391,897	35,860	0.17
Pepper	76,602,241	144,062	0.19
Rice	9,586,480	13,462	0.14

In Table 4 prediction results for the training and test sets are shown for the seven model and sampling method combinations. There are three columns of data for each performance metric. The *train* and *test* columns contain values from predictions on the training and test sets, respectively. The column *test (iso)* reports values after applying isotonic regression. The NA in the *test* columns is due to the fact that the SVM classifier does not produce probability estimates for class membership. Isotonic regression was applied to the SVM decision function output to get probability estimates for the samples in the test set. The decision function gives a distance to the separating hyperplane for each sample.

**Table 4:** Training and testing results for misplaced read classification on simulated tomato data.

Classifier	Avg Precision			F1 Score			Brier Score		
	Train	Test	Test (iso)	Train	Test	Test (iso)	Train	Test	Test (iso)
RULR	0.718	0.718	0.710	0.150	0.151	0.151	0.1460	0.1460	0.0008
RUSVM	0.820	0.817	NA	0.182	0.183	NA	0.0006	NA	0.0006
RULRS	0.782	0.781	0.773	0.659	0.652	0.652	0.0014	0.0014	0.0007
RUSVMS	0.833	0.829	NA	0.766	0.765	NA	0.0005	NA	0.0005
DTBG	0.779	0.739	0.736	0.191	0.190	0.190	0.0107	0.0107	0.0006
ADABST	0.858	0.852	0.846	0.791	0.783	0.783	0.2480	0.2480	0.0005
RUBST	0.720	0.723	0.741	0.732	0.728	0.728	0.2160	0.2160	0.0007

After applying isotonic regression, class membership was predicted by thresholding the probability estimates at the 50% level. Reads having a misalignment probability greater than 50% were assigned to Class 1, and the remainder assigned to Class 0.

Peak performance for SVM-based models was attained with the parameter *C* set to 10. *C* was the only parameter that was varied. Kernel-type was fixed to the Radial Basis Function while the parameter *γ* was set to the reciprocal of the product of the number of features and the variance of all feature values. For ADABST, the optimal parameter values were when n_estimators was set to 2,000 and learning rate was set to 1.0 (Table S2).

The two best performing models were RUSVMS and ADABST, with ADABST giving slightly better results across all three metrics. These models were used to calculate the same metrics on predictions made against the tomato, pepper and rice data sets. ADABST performed better in rice and tomato, and had slightly higher AV, with similar Brier scores. However, RUSVMS had a slight advantage for the pepper data set. ADABST was used to re-calibrate the MAPQ scores in the three alignment files (Table 5).

**Table 5:** Misplaced read classification results for the top two models on three simulated data sets.

Classifier	Average Precision		F1 Score		Brier Score	
	Test	Test (iso)	Test	Test (iso)	Test	Test (iso)
Tomato						
RUSVMS	0.836	NA	0.765	NA	0.0005	NA
ADABST	0.854	0.847	0.785	0.785	0.2479	0.0005
Pepper						
RUSVMS	0.902	NA	0.818	NA	0.0005	NA
ADABST	0.821	0.814	0.785	0.785	0.2480	0.0006
Rice						
RUSVMS	0.757	NA	0.716	NA	0.0006	NA
ADABST	0.79	0.783	0.757	0.757	0.2480	0.0005

**Figure 3:**
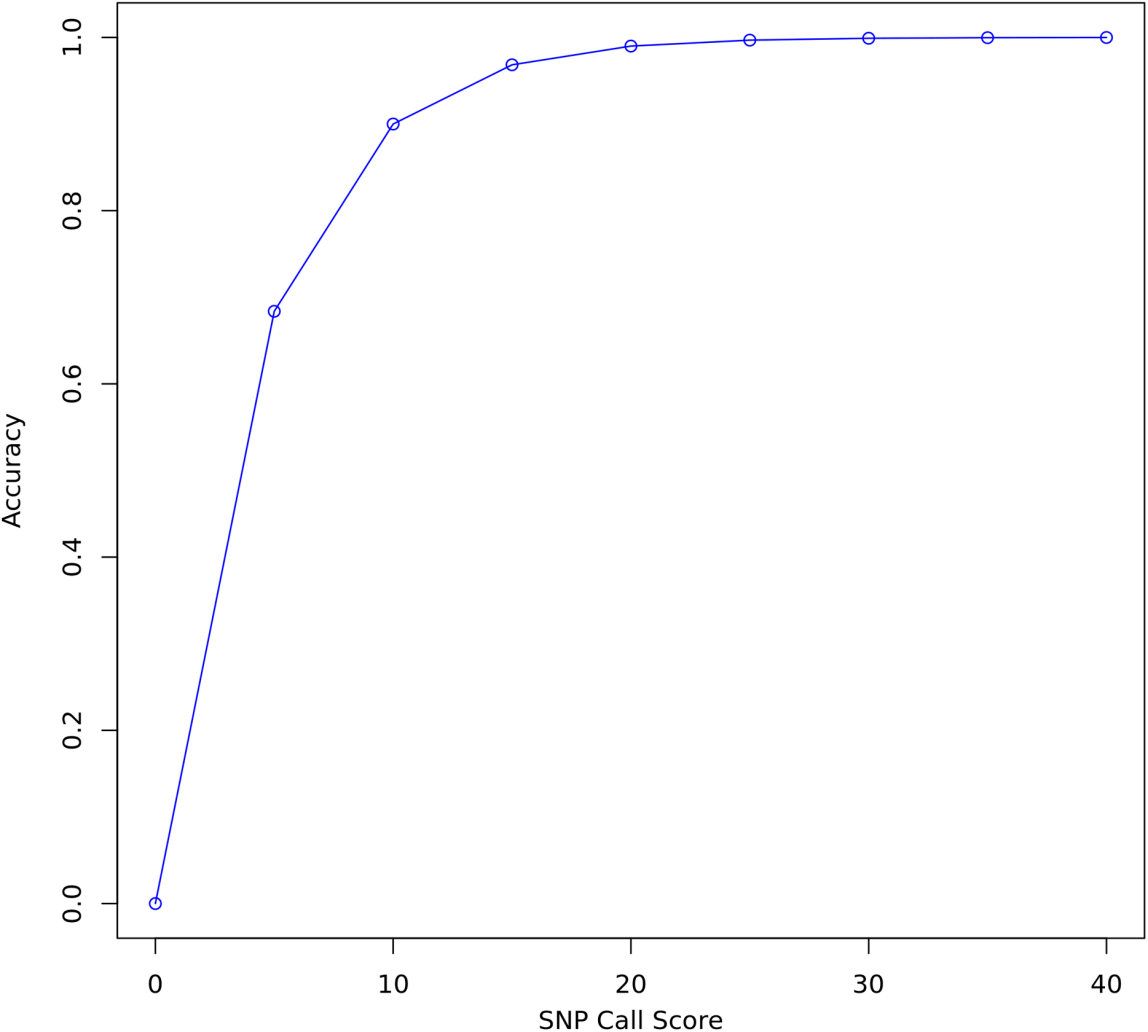
SNP call accuracy plotted as a function of call quality score.

FreeBayes and Bcftools were used to call variants on all three data sets before and after re-calibration. FreeBayes emits a quality score for each polymorphism it detects. A threshold of 20 was chosen for separating confident calls from calls likely to be due to sequencing error. Twenty is the recommended minimum in the FreeBayes manual and represents where the plot of accuracy vs score begins to flatten out (Fig. 3). A score of 20 indicates a 1 in 100 chance of no polymorphism, that is, that a variant is a false positive. Only true and false positive SNP calls with a quality score above 20 were considered for evaluating the quality of re-calibration performance. INDELs and SVs were not considered.

Table 6 shows counts for true and false positive SNP called with FreeBayes and Bcftools and having quality score above 20. Totals are reported for both calibrated (column *cal*) and uncalibrated (column *nocal*) alignment files. The last column lists total SNPs implanted into the simulated genomes.

**Table 6:** SNP calling results on three simulated data sets.

	TP (*Q* > 20)		FP (*Q* > 20)		Total SNPs
	Nocal	Cal	Nocal	Cal	
FreeBayes					
Tomato	106,374	109,688	595	559	200,957
Pepper	200,634	203,728	1,168	1,279	274,845
Rice	14,859	15,631	141	142	33,583
Bcftools					
Tomato	96,553	104,618	504	473	200,957
Pepper	186,196	198,462	348	299	274,845
Rice	14,709	15,850	25	13	33,583

### Model performance on real data

The model described in preceding sections performed poorly on the real cucumber alignments. Over 20% of the aligned reads were predicted as misaligned and SNP calling results were significantly worse after re-calibration. However, the cucumber data differed from the tomato data in two significant ways. First, variant density was much higher. Calling SNPs on the 41× coverage data yielded 658,583 SNPs and 233,044 INDELs, for approximately one small variant every 292 bp. The simulated tomato genome had 270,000 SNPs and INDELs implanted into it, for about one variant every 3,000 bp. Second, mean fragment length and its standard deviation were larger. In the cucumber alignments, mean fragment length was 390 bp with a standard deviation of 100 bp compared to 240 bp and 80 bp, respectively, in the original simulated tomato alignments.

A new set of simulated cucumber reads was generated with a similar variant density, mean fragment length and standard deviation as the cucumber data and aligned with Bowtie2. With the increased SNP density and different fragment length parameters, misplaced reads increased to 2.1% of the total.

An AdaBoost model was trained on 20% of this data and tested on the remaining 80%. The F1 score for predicting misaligned reads was 0.778, with average precision at 0.61 and a Brier score of 0.0062. The model was used to predict misplaced reads in the cucumber data and to evaluate MAPQ score recalibration effects on SNP calling. To do this, the 41× coverage cucumber data was repeatedly downsampled to 3×. The samtools view command with the -s option was used to generate 14 subsets of the cucumber data. The SNP calls on the 41× cucumber data set were used as ground truth for the comparison of SNP call results before and after MAPQ score recalibration. The SNPs and INDELs implanted into the cucumber reference were obtained by calling variants against one of the downsampled subsets of the 41× coverage cucumber data.

The model predicted 6.1% of reads as misaligned in the entire 41× data set. This is much higher than the 2.1% actually misaligned in the simulated cucumber data set. However, using the predictions to compute new MAPQ scores resulted in improvements in SNP calling performance. The average number of true and false positive SNP calls for the fourteen 3× cucumber subsets is shown in Table 7.

**Table 7:** SNP calling results on cucumber data.

	TP (*Q* > 20)		FP (*Q* > 20)		Total SNPs
	Nocal	Cal	Nocal	Cal	
FreeBayes	337,779	350,612	3,597	4,104	658,563
Bcftools	324,405	345,105	4,165	4,572	658,563

**Figure 4:**
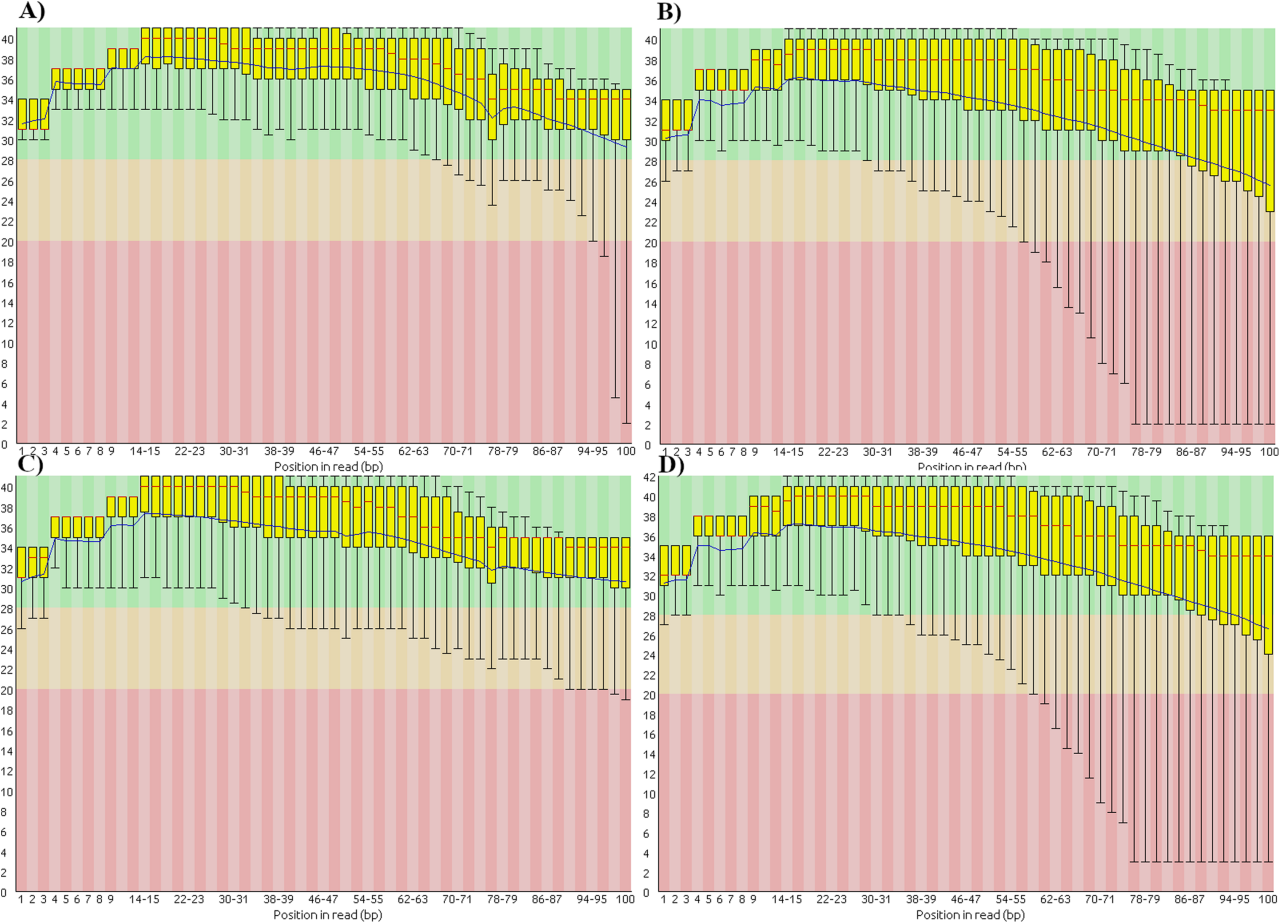
Base-quality score distributions for real and simulated data sets. (A) Real tomato data. (B) Real cucumber data. (C) Simulated tomato data. (D) Simulated cucumber data.

We also found that the base-call quality profiles of real and simulated data sets can differ significantly (Fig. 4). In the tomato data sets the default Illumina HiSeq 2000 profile provided by the ART read simulator tracked quite well the actual profile in the real tomato data (Figs. 4A and 4C). This was not the case for the cucumber data. The default profile in ART will produce base-quality score distributions in Fig. 4C. The actual distributions in the cucumber data are shown in Fig. 4B.

ART provides a script to generate base-quality score profiles from sets of FASTQ files. With that tool we generated a profile from the downsampled cucumber data and used it in the cucumber data simulation. Figure 4D shows the base-quality score distributions in the simulated cucumber data. They match very well the distributions in the real data.

All of this leads to a simple protocol for re-calibrating the MAPQ scores in real data sets:

1. Map real data reads with Bowtie2
2. Call variants with either FreeBayes or BCFTools
3. Generate an ART quality profile from the reads in FASTQ format
4. Create simulated reads using the called variants and generated quality profile
5. Train an AdaBoost model on the simulated data
6. Predict misaligned reads with the trained model
7. Use predictions to create a re-calibrated BAM file

We provide a a script that implements this in the GitHub repository. Note that for training the AdaBoost model, we simply use all the features shown in Table 1 rather than trying to select only the top few features as those can be different for simulations with different variant densities and mean fragment lengths.

## Discussion

Our study investigated whether MAPQ score re-calibration could improve the results of SNP detection in low-coverage data. We used simulated plant genomes to generate simulated sequencer reads and aligned them to the original, unaltered reference genomes. Machine learning was used to predict misaligned reads and re-calibrate estimates of misalignment probability. These estimates were used to compute new MAPQ scores and create updated SAM files for the three simulated genomes. SNPs were then called before and after re-calibration and the results compared.

Bowtie2 aligned just over 99% of the simulated reads to the original reference genomes. After excluding multi-mapped reads, misaligned reads comprised 0.14–0.19% of the total number of reads (Table 3). This increased to 2% for the reads generated in the second tomato genome simulation. This seems to have more to do with the larger mean fragment size and standard deviation than the increased variant density.

Isotonic regression improved the calibration of probability estimates produced by all classification and sampling combinations, albeit at a small cost to classifier performance. It significantly improved Brier scores, but slightly lowered AV scores (Table 4).

ADABST and RUSVMS performed best. They were used to predict misaligned reads in the simulated data sets for all three organisms. ADABST gave the best overall classification performance for tomato and rice. AV and F1 scores were higher than for RUSVMS while the Brier score was lower. RUSVMS performed better on the pepper data set. Overall, RUSVMS performed nearly as well as ADABST (Table 5). This is interesting as the number of training examples was very small (Table S3), only 302 samples compared to about 4.5 million samples to train ADABST. It is surprising that such a tiny fraction of the data set worked so well for the RUSVMS method. While ADABST performed slightly better, training time was 14 h for the entire, unbalanced training set. RUSVMS required only 5 h. Each method utilized 58 CPU cores. RULR, RUSVM and DTBG all performed poorly in terms of their respective F1 scores. RULR and RUSVM used simple RUS to combat the class imbalance in the training data. The modified RUS methods, RULRS and RUSVMS, performed considerably better in terms of both F1 and AV scores. This comes at a cost in training time as the extra steps of isolating true and false positive calls and retraining the model at every iteration is expensive.

Total true positive SNP calls increased after re-calibration. For all three data sets, the number of true SNPs detected is significantly less than the number implanted into the original reference genomes (Table 6). This is to be expected as coverage was slightly less than 2.8 for all three data sets.

Table 8 lists precision and recall figures for all three simulated data sets, before and after MAPQ score re-calibration. For the SNP calls performed by FreeBayes, in tomato there was a 3.1% increase in recall for SNP calls with a score above 20. In pepper and rice there was an improvement in recall by 1.55% and 5.18%, respectively. The numbers are greater for the Bcftools calls. For tomato there was an 8.3% increase in recall for SNP calls with a score above 20. In pepper and rice there was an improvement in recall by 6.6% and 7.7%, respectively. In all cases precision changed by only a fraction of a percent.

**Table 8:** Precision and recall for SNP calls with *Q* > 20.

Organism	Precision		Recall		δ Precision (%)	δ Recall (%)
	Nocal	Cal	Nocal	Cal		
FreeBayes						
Tomato	0.9944	0.9949	0.5293	0.5458	0.05	3.12
Pepper	0.9942	0.9938	0.7299	0.7412	−0.04	1.55
Rice	0.9906	0.9910	0.4425	0.4654	0.04	5.18
Bcftools						
Tomato	0.9948	0.9954	0.4805	0.5206	0.06	8.32
Pepper	0.9981	0.9985	0.6775	0.7221	0.04	6.58
Rice	0.9983	0.9992	0.4380	0.4720	0.09	7.76

It is clear that FreeBayes is a more permissive caller than Bcftools in that it reports far more polymorphisms, though most of them have very low quality scores. We adjusted the input parameters for Bcftools to be as permissive as possible so as to more clearly see the effects of MAPQ score re-calibration on SNP calling. By default, Bcftools ignores anomalous and overlapping read pairs and performs realignment to compute Base Alignment Quality (BAQ) scores. All of these parameters reduce the number of polymorphisms detected by Bcftools. These options were disabled for our study.

**Figure 5:**
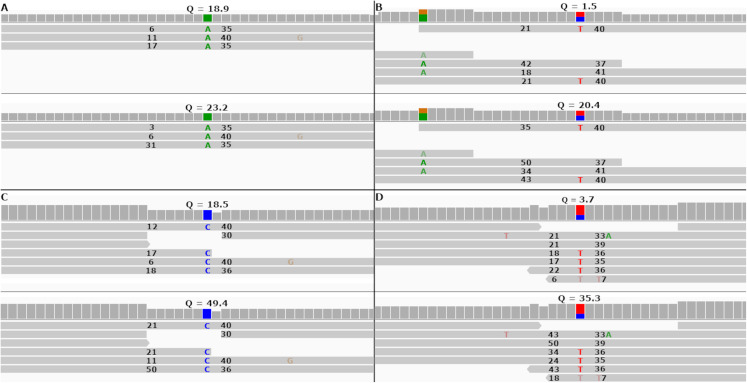
Re-calibrated mapping quality scores change SNP call quality scores. The figure shows homozygous and heterozygous SNPs embedded into the simulated tomato genome. The top half of each sub-figure shows the SNP quality score (*Q*), MAPQ scores (to the left of the indicated SNP) and base quality scores (to the right of the indicated SNP) before re-calibration. The bottom half shows the same numbers after re-calibration. Each SNP call was assigned a quality score under 20 before re-calibration and over 20 after re-calibration. (A and B) SNP calls made by FreeBayes. (C and D) SNP calls made by Bcftools.

FreeBayes can be set to take into account MAPQ scores by using the—use-mapping-quality command line parameter. The FreeBayes manual recommends against using this option unless the user knows the MAPQ scores are well calibrated. With this option enabled, FreeBayes will use in its calculations the lesser of MAPQ or base call quality scores. Figures 5A and 5B show two SNPs in the simulated tomato data called by FreeBayes.

In both cases FreeBayes assigned a SNP call quality score higher than 20 after re-calibrating the MAPQ scores. The same SNPs called without the—use-mapping-quality parameter gave SNP call quality scores 18.9 and 1.5 before recalibration, as in Figs. 5A and 5B. Post-calibration those scores increased to 34.4 and 14.2, respectively. This shows that even without the—use-mapping-quality command line parameter, FreeBayes still considers MAPQ in some way. Bcftools also utilizes MAPQ in its calculations. This can be clearly seen by the change in MAPQ scores shown in Figs. 5C and 5D.

**Figure 6:**
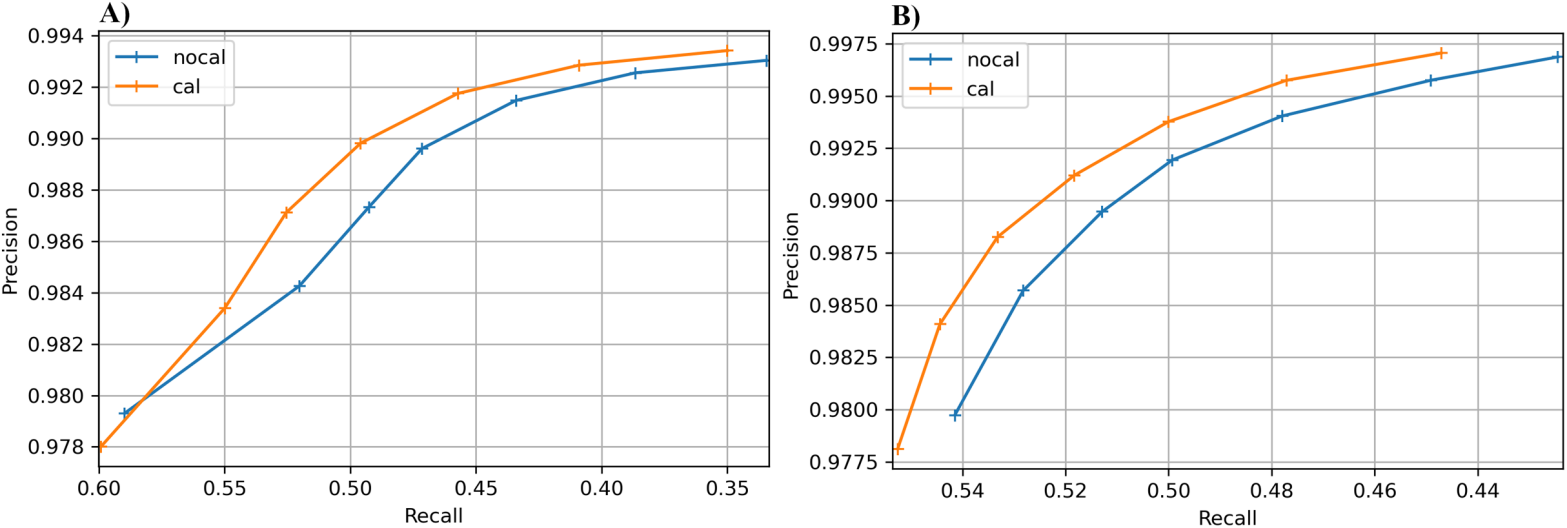
Precision-recall curves for SNP calling results. Precision vs recall is plotted for seven different SNP-quality score thresholds (10, 15, 20, 25, 30, 35, 40) marked by “+” signs on the plot. The thresholds are plotted in increasing order from left to right. (A) BcfTools SNP calls. (B) FreeBayes SNP calls.

The results for the real cucumber data show similar percentage increases in recall, with very small decreases in precision (Table 9). Figure 6 shows precision-recall curves for BcfTools (part A) and FreeBayes (part B). Precision and recall are plotted for seven SNP quality-score levels. For both variant callers we see a clear improvement in recall at almost every quality threshold, with improvements in both recall and precision at the higher quality thresholds.

**Table 9:** Averages of precision and recall for 14 3× coverage cucumber SNP calls with *Q* > 20.

Caller	Precision		Recall		δ Precision (%)	δ Recall (%)
	Nocal	Cal	Nocal	Cal		
FreeBayes	0.9895	0.9884	0.5129	0.5324	−0.10	3.78
Bcftools	0.9873	0.9869	0.4926	0.5240	−0.04	6.38

An interesting point is that classifier performance on the rice data set is better than on tomato and pepper. Both the tomato and pepper genomes contained similar numbers of SNPs and INDELs. The rice genome had around 12% of the number of variants implanted into it compared to pepper and tomato. Additionally, classification performance was tested on another simulated pepper genome with a variant density of one per 428bp. Classifier performance was comparable to the results presented for rice.

The study by Yang et al. (2015) generated 14 sets of simulated reads. Each set had a different number of SNPs implanted into the reference genome. Those numbers varied from 0.1% to 10% of the total number of nucleotides in the reference genome. Classifier performance dropped dramatically as the number of SNPs increased. Indeed, in their article the authors state that the ability of their model to identify incorrectly mapped reads improved as the number of SNPs implanted into the reference decreased. This was also the case in our study. In the second simulated tomato data set, increased variant density clearly had an effect on classifier performance. The F1 score was on par with the other simulations, but the average precision score slipped to 0.6 from over 0.8 in the other simulated data sets.

The authors also said they generated enough simulated reads to get a total of 10,000 misaligned reads and paired those with 10,000 correctly aligned reads to build their training set. This gives a hint that they recognized that their data set suffered from class imbalance. Clearly, they used a random under sampling scheme to build the training set, even though it is not explicitly mentioned in their article.

It is unlikely that filtering out misaligned reads has a big effect on variant calling performance. In most cases the number of misaligned reads is several orders of magnitude lower than the number of correctly aligned reads. Therefore, misaligned reads are unlikely by themselves to have a large impact on SNP detection.

The study by Ruffalo et al. (2012) does not mention the number of incorrect and correct alignments used for training, or how many of each were present in their data. The authors also do not state their method for evaluating their selected features or provide any indication of how informative they are.

Our study included all the features used by Ruffalo et al. (2012). Several were found to be informative and were included in the features selected for this work. They used the slope, intercept and *R* value of the least-squares line for base quality vs position in read position to characterize overall read quality. In our study these features did not have any predictive value. We also found that logistic regression was not sufficient to accurately predict incorrect alignments and adequately re-calibrate MAPQ scores.

## Conclusion

Well calibrated MAPQ scores can improve the results of downstream analyses that use those scores in their algorithms. While the gains are modest for a single sample, re-calibrating all the alignments in a multi-sample experiment might lead to larger gains. In fact, this will be the focus of a future experiment.

This study relied mostly on simulated data. While care was taken to ensure that the simulated genome used for generating training data was as realistic as possible, it was not a real assembly of a real organism. The ART read simulator does not simulate PCR bias, which affects the distribution of nucleotides in the 5′ ends of reads. Substitution errors in Illumina sequencing are known to be partially dependent on preceding nucleotides. ART does not simulate this and instead relies on profiles of substitution probabilities per nucleotide type. Also, long insertions were not included in the simulation of SVs.

We focused on a single read mapper. We did try to use BWA (Li & Durbin, 2009), but were unable to produce a model that gave good results predicting misaligned reads. Interestingly, BWA produces fewer than half the misaligned reads Bowtie2 does (using default parameters for both). This exacerbates the class imbalance problem. Also, there are fewer distinct MAPQ scores and they are not as predictive of correct alignment as those produced by Bowtie2. One of the previous studies we cite used BWA and trained their models on a balanced subset of the training data. We found this did not generalize at all to the full, imbalanced data sets. We therefore excluded BWA from our study. A more exhaustive effort to characterize the read placement behavior of BWA could be an interesting project for future investigation.

For the simulation part of our study we trained our models only on the simulated tomato data and used them to predict misaligned reads in the simulated data of other organisms, and this worked well for the three simulated data sets. Even in the presence of severe class imbalance we obtained reasonably accurate predictions and were able to demonstrably improve the calibration of MAPQ scores. This led to improved SNP detection results as shown by the increase in recall without a loss in precision. However, those three data sets had identical mean fragment lengths and standard deviations and very similar base-quality score distributions.

The real cucumber data had a larger mean fragment length and standard deviation. Variant density was considerably higher and the base-quality score distributions did not match well the distributions produced by ART’s default quality profile. We developed a simple protocol for simulating cucumber reads with parameters that match closely the real data. Using the model trained on the simulated cucumber alignments to re-calibrate MAPQ scores resulted in SNP calls that showed a definite improvement recall without a significant loss of precision. A future study will investigate how well the techniques developed here generalize to real data sets of other organisms.

## Supplemental Information

10.7717/peerj.10501/supp-1 Supplemental Information 1 Features used for training machine learning models.

10.7717/peerj.10501/supp-2 Supplemental Information 2 Parameters and values used in grid search. The parameters in the table are as defined in the *sklearn* machine learning Python package. Parameter definitions and possible values can be found in the *sklearn* user manual.

10.7717/peerj.10501/supp-3 Supplemental Information 3 Number of training examples used in each model and sampling method.

10.7717/peerj.10501/supp-4 Supplemental Information 4 Flowchart depicting the genome simulation process. Input variants are supplied in VCF format and the reference genome in FASTA format. The simulated genome is output in FASTA format. A VCF file containing all implanted variants is output as "ground truth".

10.7717/peerj.10501/supp-5 Supplemental Information 5 Flowchart detailing the feature selection process.

10.7717/peerj.10501/supp-6 Supplemental Information 6 The random under-sampling procedure flowchart.

10.7717/peerj.10501/supp-7 Supplemental Information 7 Random under-sampling of true and false positives.

10.7717/peerj.10501/supp-8 Supplemental Information 8 Links to data and usage instructions for programs and scripts.

## Additional Information and Declarations

### Data Availability

The following information was supplied regarding data availability: Source code, data and links to data are available at GitHub: https://github.com/bioinfo2019/qual-recal.
