# Supplementary figures and images for: Recalibration of mapping quality scores in Illumina short-read alignments improves SNP detection results in low-coverage sequencing data
Source: PeerJ. 2020 Dec 7;8:e10501. doi: 10.7717/peerj.10501 (PMC7727374; doi:10.7717/peerj.10501)

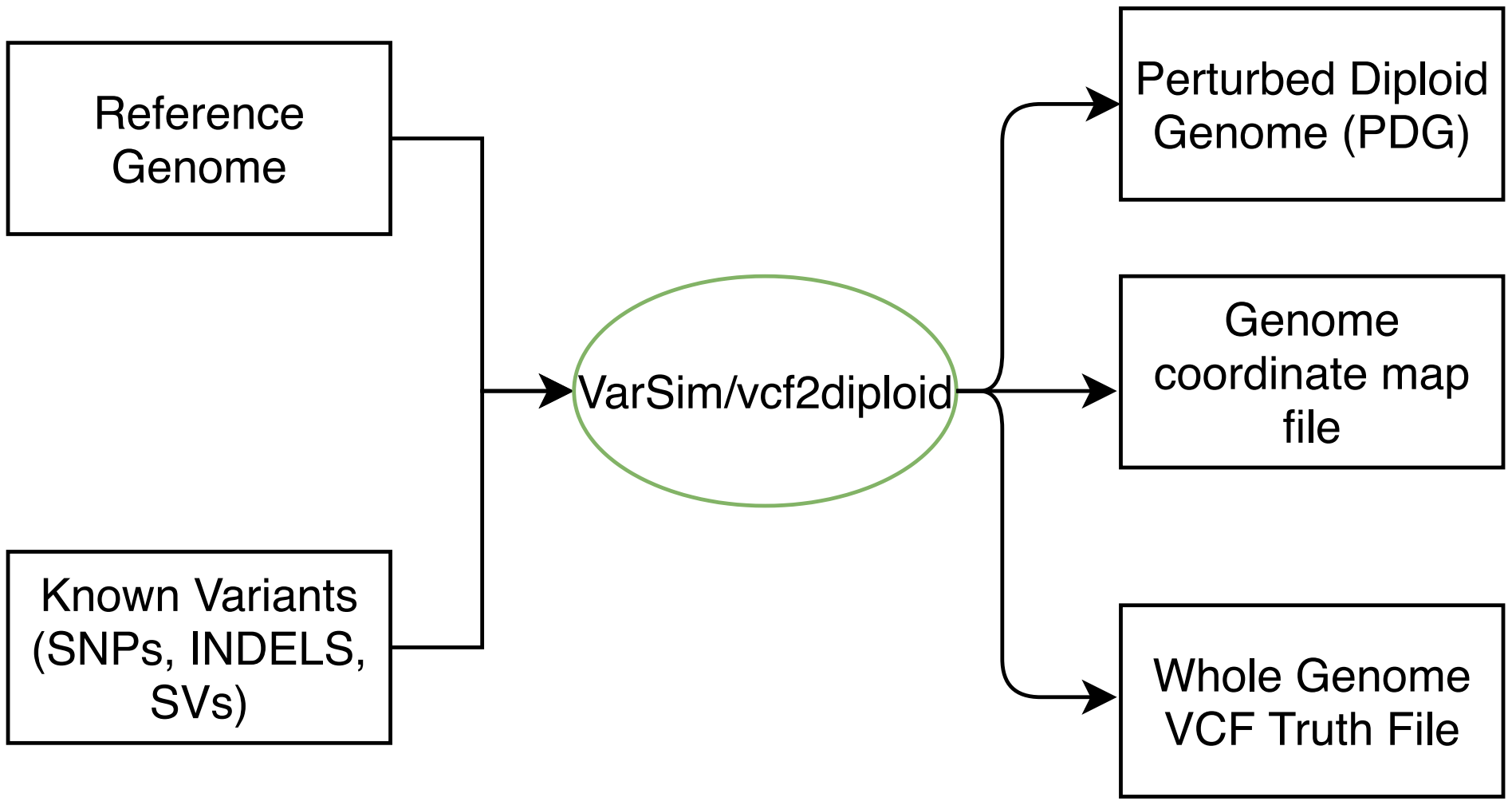

Supplement: Supplemental Information 4 — Input variants are supplied in VCF format and the reference genome in FASTA format. The simulated genome is output in FASTA format. A VCF file containing all implanted variants is output as "ground truth". [file peerj-08-10501-s004.png]

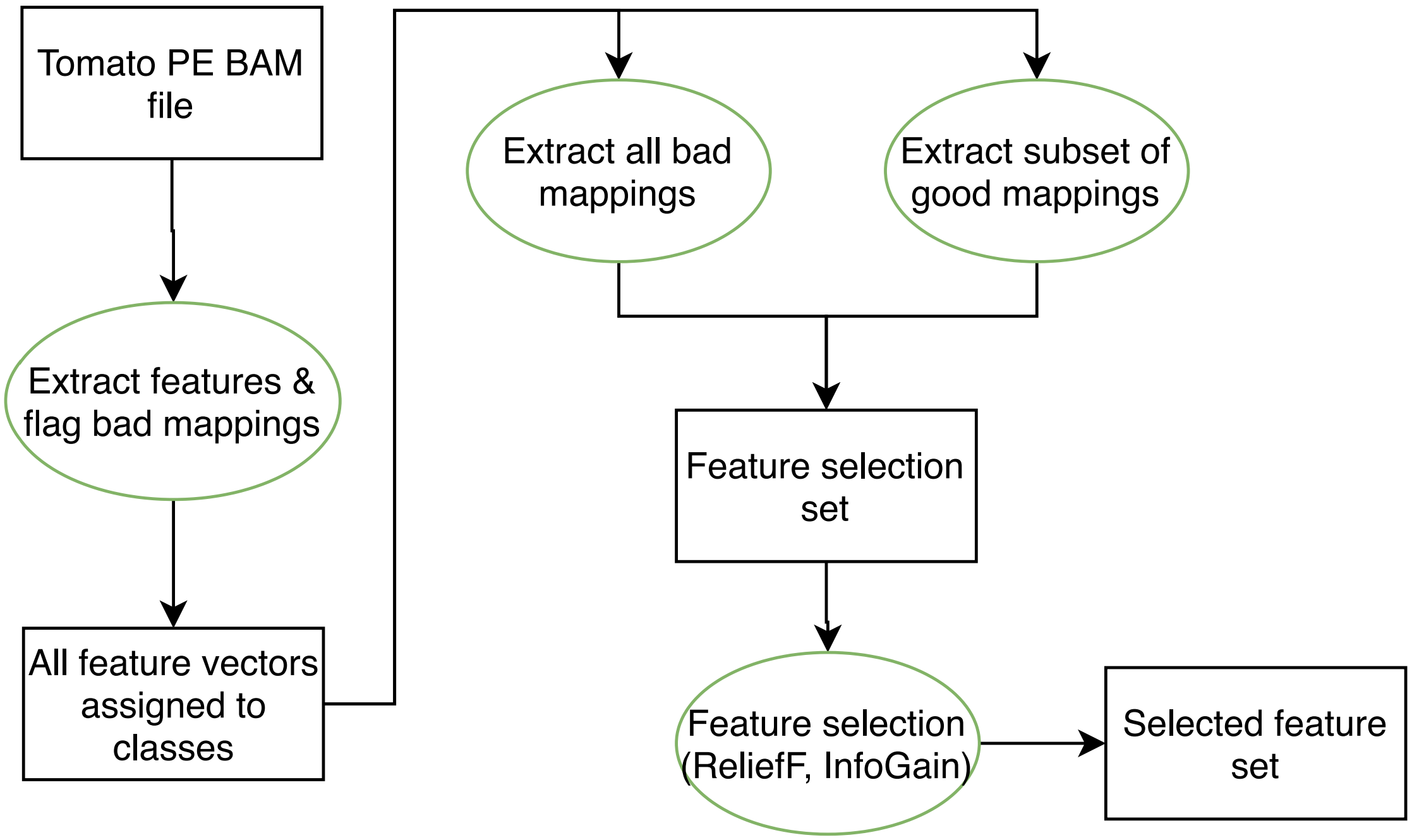

Supplement: Supplemental Information 5 [file peerj-08-10501-s005.png]

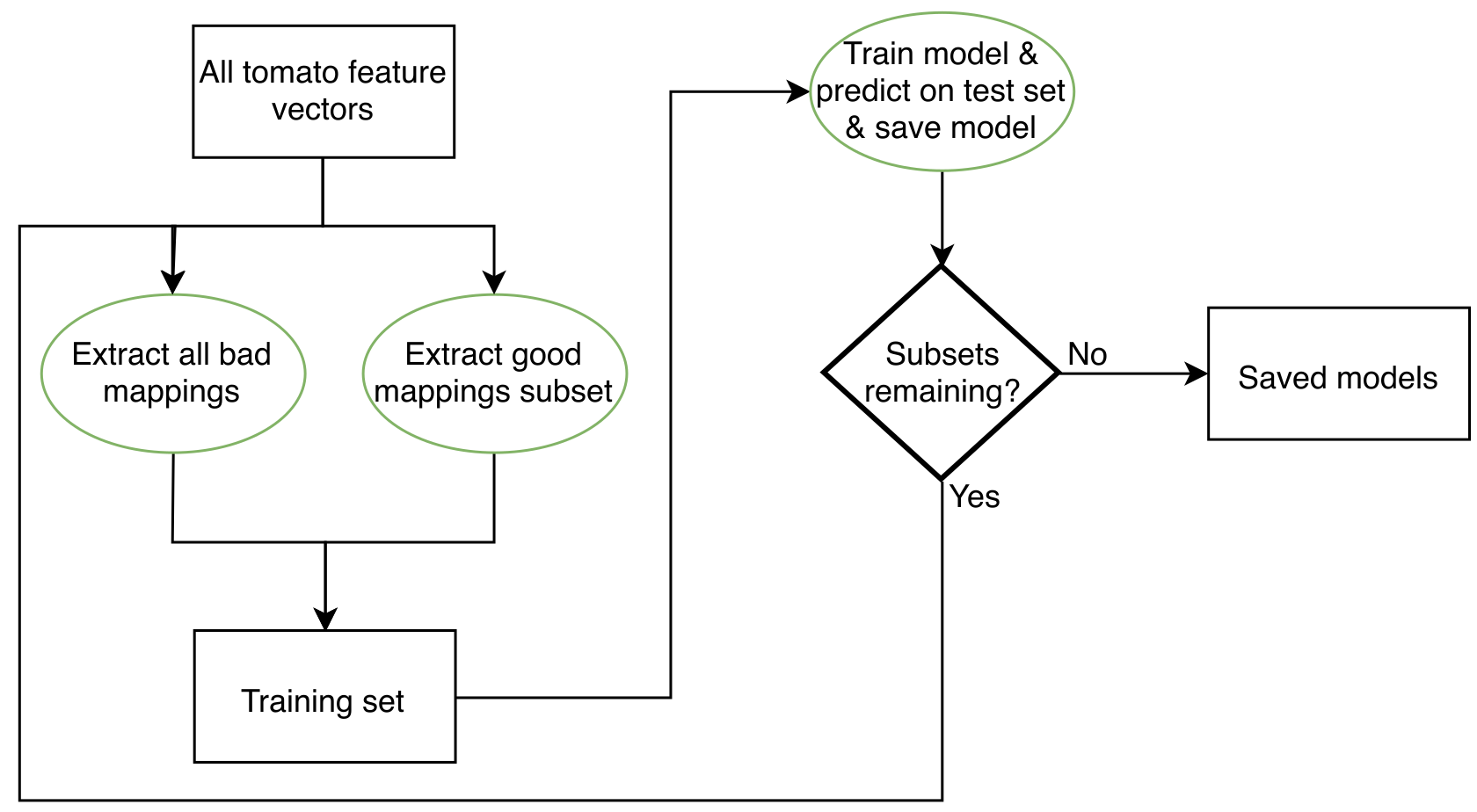

Supplement: Supplemental Information 6 [file peerj-08-10501-s006.png]

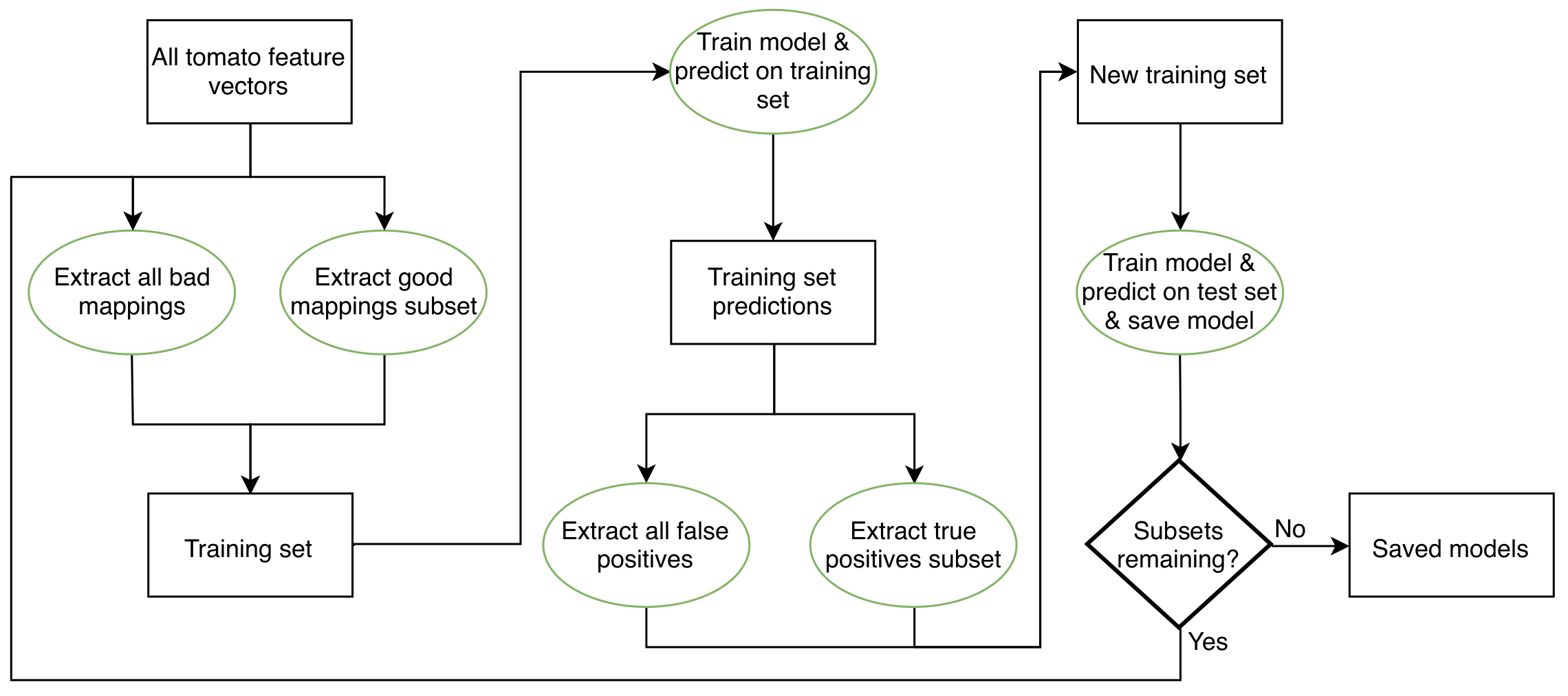

Supplement: Supplemental Information 7 [file peerj-08-10501-s007.png]
