# Supplementary material for: Recalibration of mapping quality scores in Illumina short-read alignments improves SNP detection results in low-coverage sequencing data
Source: PeerJ. 2020 Dec 7;8:e10501. doi: 10.7717/peerj.10501 (PMC7727374; doi:10.7717/peerj.10501)
Supplement: Supplemental Information 8 [file peerj-08-10501-s008.pdf]

Recalibrating Probability Estimates Used to Compute Mapping Quality Scores

The Github repository at https://github.com/bioinfo2019/qual-recal contains programs, scripts and data for investigating the effects of mapping-quality score recalibration on variant detection in low-coverage, whole-genome DNA sequencing data.

The resources in the repository allow for the simulation of diploid plant genomes implanted with variants such as SNP, INDEL and various structural variants. Simulated paired-end reads can be generated from these simulated genomes with the ART read simulation tool. Mapping the simulated reads with Bowtie2 back to their original reference genomes will create BAM files needed for the analysis pipeline described below.

Software

All software is run in Linux (Ubuntu 18.04). The pipeline here **IS NOT** a production-ready tool. These are scripts and programs written to answer a particular research question. Development of a high-performance version of these scripts and programs is underway.

The C++ folder in this repository contain programs to perform functions for extracting features from SAM (sequence/Alignment Map) files to use in training machine learning models to detect incorrectly aligned reads. The two files in the *bamlib* subfolder depend on the SeqAn sequence analysis C++ library.

https://www.seqan.de/

Those two files should be compiled as a shared object library using the C++14 dialect. The files in the *recal* subfolder should be compiled as an application that links to the bamlib shared object. The *main* routine can be found in the file nrmap.cc.

A GPU is used to compute the tens of millions of least squares lines and correlations of the base call quality scores vs. position in read. A CUDA compute level 6.1 GPU must exist with the CUDA 10.1 libraries installed.

The programs in the Python folder train models, implement data sampling schemes and make predictions on unseen samples.

Besides the resources here, a few external tools are needed. The VarSim diploid genome simulation package is available below:

https://github.com/bioinform/varsim/releases/download/v0.7.1/varsim-0.7.1.tar.gz

Setup VarSim as described in the user manual. VarSim utilizes the ART Illumina read simulator. Get the latest (Mount Rainier) version here:

https://www.niehs.nih.gov/research/resources/software/biostatistics/art/index.cfm

Bowtie2 version 2.3.5.1 is used as the read mapper, and Samtools and HTSLib version 1.9 needs to be installed as well.

http://bowtie-bio.sourceforge.net/bowtie2/index.shtml

http://www.htslib.org/download/

Variant calling was performed with both FreeBayes version 1.3.0 and Bcftools version 1.9 (part of the Samtools suite). They can be obtained from here:

https://github.com/ekg/freebayes

http://bowtie-bio.sourceforge.net/bowtie2/index.shtml

Data

Data needed to replicate the results in our paper include the tomato (S. lycopersicon) genome assembly version SL2.50, pepper (C. annuum) version 1.55 and rice (Oryza sativa L. ssp.indica) version ASM465v1:

ftp://ftp.solgenomics.net/genomes/Solanum_lycopersicum/assembly/build_2.50/S_lycopersicum_chromosomes.2.50.fa.gz

ftp://ftp.solgenomics.net/genomes/Capsicum_annuum/C.annuum_cvCM334/assemblies/Pepper.v.1.55.total.chr.gz

https://www.ncbi.nlm.nih.gov/assembly/GCA_000004655.2/

VCF files containing the SNPs and INDELS used for simulating the tomato genomes can be found here:

ftp://ftp.solgenomics.net/genomes/tomato_150/150_VCFs_2.50/RF_002_SZAXPI009284-57.vcf.gz.snpeff.vcf.gz

ftp://ftp.solgenomics.net/genomes/tomato_150/150_VCFs_2.50/RF_037_SZAXPI008747-46.vcf.gz.snpeff.vcf.gz

VCF files containing the structural variants for tomato can be found in the SV folder, and VCF files containing SNPs and INDELs for rice, pepper and cucumber are also located in the VCF folder. VCF files for structural variants, SNPs and small INDELs were used as input to the VarSim program to create the simulated tomato genome, while only SNPs and INDELs were used in the simulated genomes of pepper and rice.

The genome assemblies and variants referenced above were used to create the various simulated genomes used in the paper. The final model was also tested on real cucumber data.

Sequence reads for the Chinese Long cucumber variety were obtained from the NCBI Sequence Read Archive, accession number PRJNA339498. The reads are 101bp in length and were generated by the Illumina HS2000 sequencer, the same model sequencer that is simulated in this study. The cucumber data was used to test on real-world data the models developed with simulated data. This data can be obtained from the link below.

https://www.ncbi.nlm.nih.gov/bioproject/PRJNA339498

Analysis Pipeline Overview

1. Simulate genome and reads
2. Align reads to reference with Bowtie2
3. Extract features from BAM file for model training and misalignment prediction
4. Train a ML model to detect misaligned reads and collect stats (Brier score, F1 score, Average Precision Score)
5. Predict misaligned reads – Outputs CSV file with read names and updated MAPQ score
6. Create new BAM file with updated MAPQ scores
7. Call SNPs on original and updated BAM files using FreeBayes and Bcftools
8. Compare VCF files from the SNP calling step and collect stats (True and False positive raw totals, Precision, Recall)

A few Python 3 and Bash shell scripts drive each of the steps listed above. These scripts have dependencies that are listed in earlier sections. Once those dependencies have been met, the scripts should run with no problems.

Model training can take substantial amounts of time. Grid search is used to hyper-parameter optimization. This is not very efficient, but generally yields good results. It is

trivial to change this to a randomized approach that does not exhaustively search the parameter space. Training the AdaBoost and SVM models takes anywhere from 7 to 14 hours depending on the computer used and the parameter grid size.

The scripts also expect a certain directory structure. The following directories should be created anywhere on the filesystem that is convenient.

```
.
├── misc
│   ├── freebayes
│   ├── nrmap
├── models
├── PythonProgs
├── varsim_run
    ├── alignments
    ├── ART
    │   ├── art_bin_MountRainier
    │   │   ├── Illumina_profiles
    ├── input_vcfs
    ├── log
    ├── out
    ├── reference
    ├── stats
    ├── variant_calls
    └── work
```

All Python scripts should be placed into the *PythonProgs* folder. Bash scripts go in the *varsim_run* folder. Extracted features and trained models will be saved into the *models* directory. VarSim and its utilities should be unpacked into the *varsim_run* folder. Use the latest version of ART, not the one supplied with VarSim, and unpack it into the *varsim_run* folder. The C++ program compiled in the steps above should be named nrmap and placed into the *nrmap* folder. A binary version of the program and its companion shared library are available in the GitHub repository for this project. Note this is for Linux AMD64 systems with a CUDA 6.1 capable GPU installed and the Cude 10.1 libraries on your system.

FreeBayes should be unpacked into the *misc* subfolder. Bcftools and Samtools can be anywhere on the system, but be sure they are in the system PATH variable.

Fasta genome sequences and their *.fai* indexes need to be in the *varsim_run/reference* folder as do their Bowtie2 indexes. The VCF files containing variants to be implanted into the reference genomes should be placed into *varsim_run/input_vcfs*.

Simulated reads produced by VarSim will be saved into *varsim_run/out* and variants called by the pipeline scripts will be saved into *varsim_run/variant_calls*. Totals and statistics output by the scripts will be saved to the *varsim_run/stats* folder.

At the top of each of the bash shell scripts is a variable called BASE_DIR can be set that specifies the folder under which the above directory structure was created. You can set its value like

BASE_DIR=/top/level/path

The bash script, *mapping.sh*, drives the process of

- Simulating genomes and reads
- Mapping reads
- Extracting features

Another bash script, *recal.sh*, handles

- Generating a recalibrated BAM file
- Calling SNPs with the original and recalibrated BAM files
- Comparing the resulting VCF files

The bash script *downsample.sh* takes care of

- Mapping the 41x coverage cucumber reads to the reference genome
- Calling SNPs to be used as "ground truth"
- Downsampling the 41x coverage BAM file to 3x coverage (14 times)
- Extracting features
- Predicting bad reads
- Recalibrating the BAM file
- Calling SNPs on the 3x subsets
- Comparing SNP calls before and after MAPQ score recalibration
- Writing out the statistics for each of the 3x subsets

The various Python scripts are themselves called from the bash shell scripts. They are quite straightforward and have only a couple of command line parameters.

ModelTraining.py is used to train ML and isotonic regression models. They are saved to disk using the Python pickle method. This script takes the following command line parameters:

--base-dir	the top-level directory holding the folders described above
--ml-model	the machine learning model to be trained.
--features	a comma separated list of features to be used for training
--feats-file	the text file containing the feature vectors

The list of models that can be trained and features that can be specified available in the *mapping.sh* bash script.

Model.Training.py should be run before either *mapping.sh* or *recal.sh*.

ModelPredict.py is called by the bash script recal.sh. It has the same parameters as *ModelTraining.py*. *ModelPredict.py* predicts misaligned reads from features extracted from the BAM alignment files and writes out a simple CSV file with each row containing a read name and an updated MAPQ score. This is used by the C++ program, *nrmap*, to create a new BAM file with the update MAPQ scores.

The Python script *snp_stats.py* prints to screen and writes out to a CSV files the raw true/false positive totals, precision and recall for SNP calls made before and after MAPQ score recalibration. It also outputs the percentage change in both precision and recall after recalibration. It takes only one command line parameter:

--base-dir   the top-level directory holding the folders described above

Basically, the bash scripts drive the entire process and there shouldn't be any need to use the Python scripts, or other programs, directly to duplicate the research in our paper.
